# Supplementary material for: Drug repurposing for aging research using model organisms
Source: Aging Cell. 2017 Jun 16;16(5):1006–15. doi: 10.1111/acel.12626 (PMC5595691; doi:10.1111/acel.12626)
Supplement: Supplementary file 7 — Data S1 Zip‐Archive of all report cards. [file ACEL-16-1006-s007.zip › RC_9LI.pdf]

# 9LI

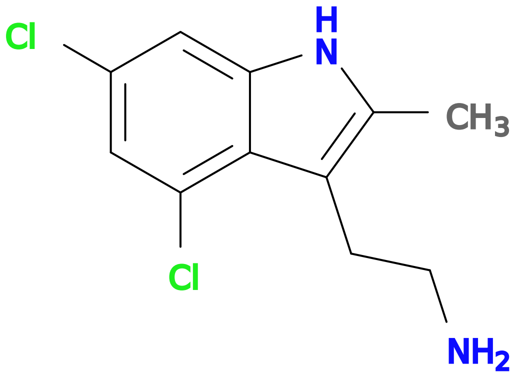


**Database identifiers**

| | |
|---|---|
| ChEMBLCompound | CHEMBL2396992 |
| eMolecules | 710467 |

## Ranking

| | Rank | Score |
|---|---|---|
| Drosophila | 421/697 | 0.34 |
| C. elegans | 368/591 | 0.162 |

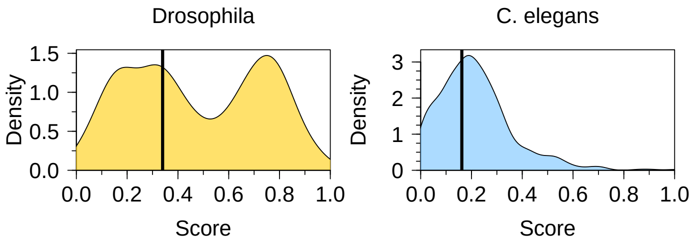


| | Ageing implication | Domain conservation | Binding site conservation | Binding affinity | Bioavailability | Lipinski | Promiscuity | Purchasability | Drug approval | **Total** |
|---|---|---|---|---|---|---|---|---|---|---|
| Drosophila | 0.81 | 0.964 | 1.0 | 0.342 | (0.9) | 0.0 | -0.0 | 0.1 | 0.0 | 0.34 |
| C. elegans | 0.81 | 0.962 | 1.0 | 0.342 | 0.235 | 0.0 | -0.0 | 0.1 | 0.0 | 0.162 |

## Names

- 9Li
- Lithium, isotope of mass 9
- Lithium-9

## Roles

ChEBI entry None has no roles

## Status

| | |
|---|---|
| Approved drug (according to ChEMBL) | No |
| Number of Rule of 5 violations | 0 |
| Binding affinity to original target in log units (RF-Score prediction) | 4.34 |
| Burns *C. elegans* bioavailability prediction | -2.97 |

## Compound Target Characteristics

### GTPase KRas

Best gene implication in ageing for this target family came from gene P01112 annotated in UniProt release 2014_02. Annotation GO subterm of 7568 (aging) was Inferred from Direct Assay

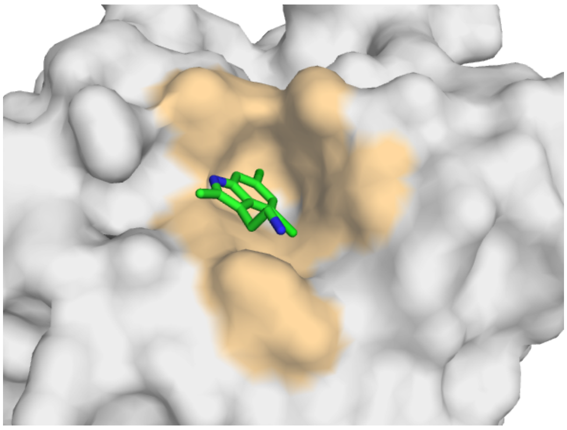

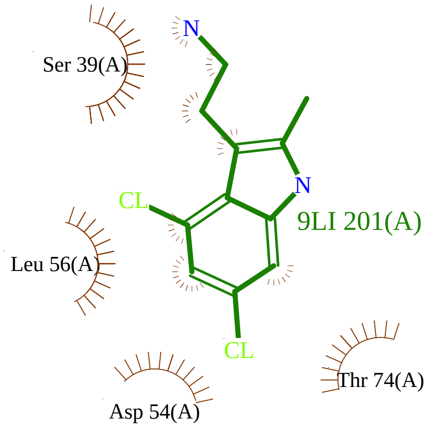


| protein | amino acids contacts (binding site) |
|---|---|
| PDB:4dst:chainA:P01116 | K V S D I L T G |
| tr:L7RSL8:L7RSL8_HUMAN | K V S D I L T G |
| sp:P01111:RASN_HUMAN | K V S D I L T G |
| tr:Q5U091:Q5U091_HUMAN | K V S D I L T G |
| sp:P01112:RASH_HUMAN | K V S D I L T G |
| sp:P01116:RASK_HUMAN | K V S D I L T G |
| tr:I1SRC5:I1SRC5_HUMAN | K V S D I L T G |
| tr:Q8CGQ3:Q8CGQ3_RAT | K V S D I L T G |
| sp:P20171:RASH_RAT | K V S D I L T G |
| sp:Q04970:RASN_RAT | K V S D I L T G |
| sp:P08644:RASK_RAT | K V S D I L T G |
| tr:A0JN17:A0JN17_RAT | K V S D I L T G |
| tr:Q71SW8:Q71SW8_MOUSE | K V S D I L T G |
| tr:P97870:P97870_MOUSE | K V S D I L T G |
| tr:C0H5X4:C0H5X4_MOUSE | K V S D I L T G |
| sp:Q61411:RASH_MOUSE | K V S D I L T G |
| sp:P32883:RASK_MOUSE | K V S D I L T G |
| tr:Q0VDV7:Q0VDV7_MOUSE | K V S D I L T G |
| tr:Q5J7N1:Q5J7N1_MOUSE | K V S D I L T G |
| tr:Q9D091:Q9D091_MOUSE | K V S D I L T G |
| tr:Q4FJP3:Q4FJP3_MOUSE | K V S D I L T G |
| sp:P08646:RAS1_DROME | K V S D I L T G |
| sp:P22981:LET60_CAEEL | K V S D I L T G |
| sp:P01119:RAS1_YEAST | K V S D I L T G |

| protein | whole protein ident | simil | domain-based ident | simil | contact-based ident | simil |
|---|---|---|---|---|---|---|
| PDB:4dst:chainA:P01116 | 0.91 | 0.96 | 0.95 | 0.98 | 1.0 | 1.0 |
| tr:L7RSL8:L7RSL8_HUMAN | 1.0 | 1.0 | 1.0 | 1.0 | 1.0 | 1.0 |
| sp:P01111:RASN_HUMAN | 0.87 | 0.96 | 0.91 | 0.97 | 1.0 | 1.0 |
| tr:Q5U091:Q5U091_HUMAN | 0.87 | 0.96 | 0.91 | 0.97 | 1.0 | 1.0 |
| sp:P01112:RASH_HUMAN | 0.86 | 0.95 | 0.9 | 0.97 | 1.0 | 1.0 |
| sp:P01116:RASK_HUMAN | 1.0 | 1.0 | 1.0 | 1.0 | 1.0 | 1.0 |
| tr:I1SRC5:I1SRC5_HUMAN | 0.64 | 0.64 | 1.0 | 1.0 | 1.0 | 1.0 |
| tr:Q8CGQ3:Q8CGQ3_RAT | 0.51 | 0.51 | 0.55 | 0.55 | 1.0 | 1.0 |
| sp:P20171:RASH_RAT | 0.86 | 0.95 | 0.9 | 0.97 | 1.0 | 1.0 |
| sp:Q04970:RASN_RAT | 0.87 | 0.96 | 0.92 | 0.97 | 1.0 | 1.0 |
| sp:P08644:RASK_RAT | 0.99 | 1.0 | 0.99 | 1.0 | 1.0 | 1.0 |
| tr:A0JN17:A0JN17_RAT | 0.89 | 0.95 | 0.94 | 0.98 | 1.0 | 1.0 |
| tr:Q71SW8:Q71SW8_MOUSE | 0.5 | 0.51 | 0.54 | 0.55 | 1.0 | 1.0 |
| tr:P97870:P97870_MOUSE | 0.51 | 0.51 | 0.55 | 0.55 | 1.0 | 1.0 |
| tr:C0H5X4:C0H5X4_MOUSE | 0.52 | 0.58 | 0.56 | 0.63 | 1.0 | 1.0 |
| sp:Q61411:RASH_MOUSE | 0.86 | 0.95 | 0.9 | 0.97 | 1.0 | 1.0 |
| sp:P32883:RASK_MOUSE | 0.99 | 1.0 | 0.99 | 1.0 | 1.0 | 1.0 |
| tr:Q0VDV7:Q0VDV7_MOUSE | 0.99 | 1.0 | 0.99 | 1.0 | 1.0 | 1.0 |
| tr:Q5J7N1:Q5J7N1_MOUSE | 0.88 | 0.95 | 0.94 | 0.98 | 1.0 | 1.0 |
| tr:Q9D091:Q9D091_MOUSE | 0.87 | 0.96 | 0.91 | 0.97 | 1.0 | 1.0 |
| tr:Q4FJP3:Q4FJP3_MOUSE | 0.85 | 0.94 | 0.91 | 0.97 | 1.0 | 1.0 |
| sp:P08646:RAS1_DROME | 0.76 | 0.91 | 0.81 | 0.93 | 1.0 | 1.0 |
| sp:P22981:LET60_CAEEL | 0.75 | 0.89 | 0.77 | 0.92 | 1.0 | 1.0 |
| sp:P01119:RAS1_YEAST | 0.35 | 0.51 | 0.6 | 0.86 | 1.0 | 1.0 |

**Ras85D (FBgn0003205) associated phenotypes**

RU486 conditional, cell autonomous, cell death defective, cell non-autonomous, cell polarity defective, cell size defective, conditional, decreased cell death, decreased cell number, decreased cell size, developmental rate defective, dominant, endocytosis defective, female semi-sterile, germline clone, heat sensitive, hyperplasia, increased cell death, increased cell growth, increased cell number, increased cell size, large body, learning defective, lethal - all die before end of P-stage, lethal - all die before end of pupal stage, majority die during P-stage, maternal effect, melanotic mass phenotype, mitotic cell cycle defective, neoplasia, neuroanatomy defective, neurophysiology defective, partially, partially lethal - majority die, short lived, size defective, small body, somatic clone, some die during P-stage, some die during pharate adult stage, some die during pupal stage, temperature conditional, terminal phenotype, tumorigenic

(Information from FlyBase)

**Ras85D (UniProt:P08646) annotation**

**Function**: May mediate a signal that determines the fate of photoreceptor cells in the developing compound eye. Ras proteins bind GDP/GTP and possess intrinsic GTPase activity.

**Enzyme regulation**: Alternates between an inactive form bound to GDP and an active form bound to GTP. Activated by a guanine nucleotide-exchange factor (GEF) and inactivated by a GTPase- activating protein (GAP).

**Subcellular location**: Cell membrane (PubMed:18503409); Lipid-anchor (PubMed:18503409); Cytoplasmic side (PubMed:18503409). Note=Loss of prenylation causes protein location to the cytoplasm.

**Tissue specificity**: Expressed in the posterior termini of the embryo, restricted mainly to the embryonic central nervous system, and in the eye imaginal disk. (PubMed:7873789).

(Information from UniProt)

**let-60 (WBGene00002335) associated phenotypes**

AWA odorant chemotaxis defective, AWC odorant chemotaxis defective, L2 arrest, L3 arrest, antibody staining reduced, antibody staining variant, axon outgrowth variant, bloated, cell differentiation variant, egg laying defective, egg laying imipramine resistant, egg laying variant, germ cell development variant, gonad arm morphology variant, lethal, locomotion variant, male mating defective, mid larval lethal, multivulva, neuron degeneration, oocyte accumulation, oocytes small,

organism starvation response variant, protein expression absent, reduced brood size, rod like larval lethal, sterile, unfertilized oocytes laid, vulval cell induction increased, vulvaless

(Information from WormBase)

**let-60 (UniProt:P22981) annotation**
**Function**: The level of let-60 controls the switch between vulval and hypodermal cell fates during C.elegans vulval induction. May stimulate the guanine nucleotide exchange factor (GEF) activity of rap-1.
**Subunit**: Interacts with soc-2. (PubMed:9674433).
**Subcellular location**: Cell membrane; Lipid-anchor.

(Information from UniProt)
